# The Role of HSP90 in Preserving the Integrity of Genomes Against Transposons Is Evolutionarily Conserved

**DOI:** 10.3390/cells10051096

**Published:** 2021-05-04

**Authors:** Valeria Specchia, Maria Pia Bozzetti

**Affiliations:** Department of Biological and Environmental Sciences and Technologies, University of Salento, 73100 Lecce, Italy; valeria.specchia@unisalento.it

**Keywords:** HSP90/HSP83, transposons, piRNA pathway

## Abstract

The HSP90 protein is a molecular chaperone intensively studied for its role in numerous cellular processes both under physiological and stress conditions. This protein acts on a wide range of substrates with a well-established role in cancer and neurological disorders. In this review, we focused on the involvement of HSP90 in the silencing of transposable elements and in the genomic integrity maintenance. The common feature of transposable elements is the potential jumping in new genomic positions, causing chromosome structure rearrangements, gene mutations, and influencing gene expression levels. The role of HSP90 in the control of these elements is evolutionarily conserved and opens new perspectives in the HSP90-related mechanisms underlying human disorders. Here, we discuss the hypothesis that its role in the piRNA pathway regulating transposons may be implicated in the onset of neurological diseases.

## 1. HSP90 Is an Evolutionarily Conserved Molecular Chaperone

The HSP90 protein exhibits an extraordinary conservation of sequence and functions during evolution and is expressed from bacteria to humans.

The evolutionary conservation and the wide expression of these proteins reflect their fundamental role in the physiology of cells. The crucial molecular role of HSP90 is to act as a chaperone. HSP90 maintains the correct folding of proteins, participates in their refold or repair, and assists in their proteasome-mediated degradation. Due to its role as a molecular chaperone, HSP90 binds and stabilizes hundreds of proteins with roles in many processes such as cellular and oxidative stress, and is also involved in cancer and neurological disorders [1,2,3,4,5,6,7,8]. This protein is expressed in physiological conditions during development and in a large number of differentiated cells, but at the same time, its expression increases during stress conditions to ensure the correct folding of misfolded targets.

HSP90 proteins have conserved domains including an amino-terminal domain (NTD) connected to a middle domain and a carboxy-terminal domain (CTD) [9]. HSP90 is an ATPase, and the ATP binding as well as hydrolysis are key features for its activity on the client proteins. In addition to the hydrolysis of ATP, the function of HSP90 is regulated by the interaction with co-chaperones. Together, ATP and co-chaperone binding ensures that a complex conformational change of HSP90 is required to become active. The co-chaperones play different roles in Hsp90. Some co-chaperones activate the ATPase activity of HPS90. Other co-chaperones are important for the loading of client protein [2,10,11,12,13,14,15]. One class of cochaperone contains a tetratricopeptide repeat (TPR) domain that binds the C-terminal domain of HSP90. This class includes evolutionarily conserved peptidylprolyl isomerases, most of which belong to the FK506-binding protein (FKBP) family. The nematode prolyl isomerase FKB-6 facilitates the formation of the complex between HSP90 and the glucocorticoid receptor (GR). Human peptidylprolyl isomerases homologs, FKBP51 and FKBP52, also support the HSP90 binding to the GR complex. The HSP90 binding to GR makes the receptor suitable for hormone binding and for transcriptional activation [16]. Moreover, HSP90/FKBP51 interacts with Tau, increases its stability [17], and promotes its neurotoxic accumulation [18].

In addition to the activities of HSP90 in hormone pathways, other roles of HSP90 on genomic DNA are also essential [19,20]. In mammalian cells, HSP90 enhances the enzyme activity of the histone methyl-transferase SMYD3, which is involved in the transcriptional regulation mediated by the RNA polymerase II (RNA pol II) [21]. In turn, Drosophila HSP90 co-operates with the Trithorax protein, a chromatin-modifying enzyme involved in gene regulation [22]. Moreover, Drosophila HSP90 acts in the nucleus, regulating the so called “pausing” of the RNA pol II, which pauses downstream of the transcription start sites blocking the transcript elongation. In the complex network of the pausing mechanism, HSP90 is involved in stabilizing the negative elongation factor (NELF), and consequently, the RNA pol II stalls on the DNA. The role of HSP90 in this relevant process of gene regulation is conserved from Drosophila to humans [19].

The crucial activities of HSP90 on the genomes also include its role in the DNA damage sensing signaling and repair [23] and its activity to avoid the deleterious jumping of transposable elements (TEs) [24,25].

## 2. HSP90 Acts to Maintain the Integrity of the Genomes

Among the processes involving HSP90, its role as a guardian of the integrity of the genomes is an interesting though not a completely explored activity. In more detail, HSP90 has been implicated in the silencing of transposable elements in different organisms [24,25,26].

### 2.1. Transposable Elements and the piRNA Pathway

**Figure 1:**
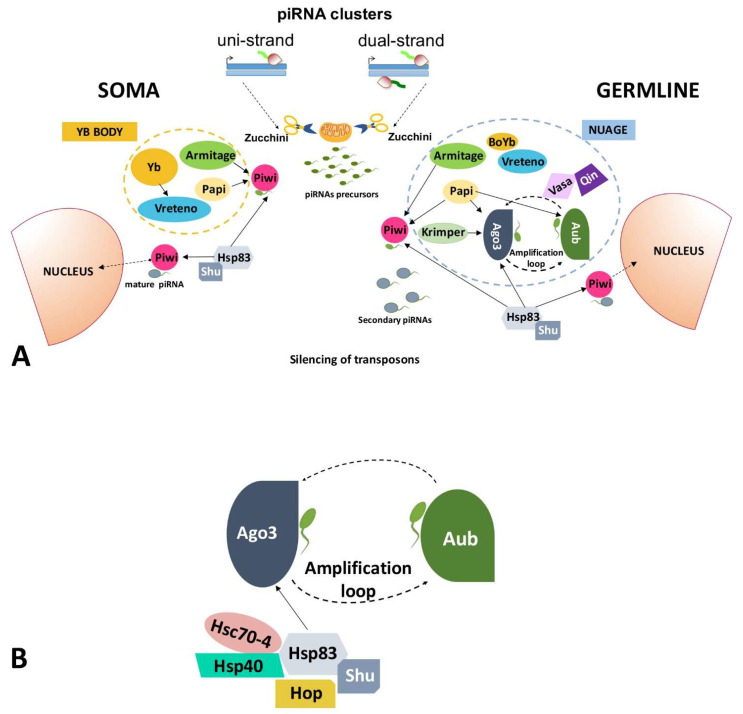
Scheme of the proteins implicated in the piRNA pathway in gonads. (**A**) Proteins involved in the gonadal piRNA are shown. The Yb body in the soma and the nuage in the germline are indicated. Arrows with a continuous line mark the interactions amongst the proteins in the soma (on the left) and in the germline (on the right). (**B**) Details of HSP83-cochaperones complex involved in the piRNA-mediated silencing of transposable elements in the germline.

Transposable elements are repetitive sequences occupying large portions of all eukaryotic genomes. These elements have been discovered by Barbara McClintock’s experiments in maize [27], and these same types of elements were identified as structural components of the heterochromatin of Drosophila [28] and humans [29]. The common peculiarity of transposable elements is the potential jumping in new genomic positions. Despite their different sequences and mechanisms of transposition [30,31,32], the effects of their movement on the genomes are common. The general insertion activity of transposons increments the gene mutation rates; at the same time, these repetitive sequences can induce chromosome structure rearrangements by recombination between multiple copies of a transposable element [33]. Furthermore, transposons modify gene expression providing promoters, cis regulatory element at promoters and enhancers, or altering the chromatin state [34,35,36,37]. The global effect of transposons on the genome is particularly deleterious for the germ cells, which must ensure the genetic material transmission to the new generations for the maintenance of the species. For this reason, a fine mechanism to block transposons has been evolved in germ cells. First discovered in *Drosophila*, this mechanism of silencing involves a specific class of small non-coding RNAs called Piwi Interacting RNAs (piRNAs) [38]. In *Drosophila*, the piRNAs interact with a subfamily of the Argonaute proteins, the PIWI subfamily including Piwi, Argonaute 3 (Ago3), and Aubergine (Aub) [39,40,41,42,43,44]. piRNAs arise as long transcript precursors from specific genomic clusters, which contain sequences of transposons and their relics [45,46,47]. The clusters, divided in uni-strand and dual-strand clusters, participate in the primary pathway that occurs in both somatic and germ cells of the ovaries. In Figure 1 and Table 1, the main components of the germline and somatic piRNA pathway, their localization, interactions, and function, are shown.

#### 2.1.1. piRNA Pathway in Germ Cells of the Ovary

In germ cells, the primary piRNAs undergo a secondary pathway, the ping-pong cycle, in which Aub and Ago3 ensure the amplification in a number of these small RNAs. The ping-pong cycle occurs in the perinuclear region or nuage of the germ cells [45,48,51]. The recruitment of the PIWI proteins to the nuage and the assemblage of the piRNA pathway components require the Tudor-domain protein PAPI [58], while the E3 ligase and Tudor-domain protein Qin maintains heterotypic ping-pong between Aub and Ago3 [49]. The Tudor-domain protein Krimper associates with Ago3 and promotes the Ago3 post-translational modification and loading of piRNAs [53]. Another pathway that generates germline piRNAs is the phased piRNA pathway producing strings of tail-to-head piRNAs. The production of phased piRNAs takes place on the mitochondria and requires the endonuclease Zucchini and the RNA-binding ATPase Armitage. Specifically, Armitage links the ping-pong cycle to the phased piRNA pathway [50] (Figure 1A).

#### 2.1.2. piRNA Pathway in Somatic Cells of the Ovary

In the somatic follicle cells of the *Drosophila* ovary, piRNAs are generated by a Piwi-dependent mechanism through the primary piRNA biogenesis pathway [59]. Follicle cells present specific cytoplasmic perinuclear structures named Yb bodies, which are discrete cytoplasmic compartments taking their name from the Yb protein. In the Yb bodies, the somatic piRNA biogenesis occurs in the ovary and testis [54,60]. The Tudor domain protein Vreteno and the RNA-binding protein Yb contribute to the Piwi stability and to the Yb body formation [59]. The helicase Armitage, another component of the Yb body, and the Yb protein are needed for piRNA loading onto Piwi. When piRNAs are loaded onto Piwi, Piwi enters the nucleus in which it exerts an epigenetic regulation of transposon sequences [60,61,62,63]. Phased “tail to head” piRNAs are also produced in the somatic cells supported by Zucchini and Armitage [64]. piRNA-Piwi complex binds DNA and recruits epigenetic factors such as HP1 and the histone methyltransferase Su(var)3–9 to establish the epigenetic silencing of transposons. This transcriptional control of transposons is predominant in the somatic follicle cells [63] (Figure 1A).

### 2.2. HSP90 Has a Role in the Regulation of Transposable Elements

The first indication of the involvement of HSP90 in the silencing of transposable elements originated from the discovery of the deregulation of transposons, with consequent expression and “movement” in germ tissues of *Drosophila* hypomorphic mutants for HSP90 [24]. The reduction of HSP90 activates transposons that affect phenotypic variations in flies [24,65,66]. This mechanism adds up to the previously proposed role for HSP90 in modulating morphological variations due to its capacity to reveal cryptic genetic mutations [67]. It was then that the molecular roles of HSP90 in the piRNA pathway became important to be studied in depth.

In *Drosophila melanogaster*, HSP90 binds to Piwi, which in turn is phosphorylated and forms active complexes with piRNAs [25]. The HSP90 co-chaperone Shutdown cooperates in order to upload the piRNAs onto Piwi protein complexes [56,57] (Figure 1A,B). In mammals, FKBP6, the mouse ortholog of HSP90 co-chaperone Shutdown with the typical TRP domain necessary to bind HSP90, is required for the secondary biogenesis of piRNAs [56]. In addition, HSP90 is important for both the piRNA production and the post-transcriptional repression of transposons in mouse fetal germ cells, and this activity is independent from FKBP6 [68]. The mouse Piwi proteins, Mili and Miwi2, are the principal actors in the piRNA pathway working in fetal germ cells. These proteins bind primary piRNAs, obtained by the cut of the precursors transcribed from the piRNA clusters, cleave the transposon transcripts to generate secondary piRNAs, and mediate DNA methylation of transposons such as Line1. The reduction of HSP90 levels reduces the amount of fetal piRNAs in complexes with Mili and Miwi2, causing the activation of transposons [68]. In mammals, the HSP90 is involved in transposon control in fetal germ cells and its physiological expression is higher in the testes and the brain [69].

Interestingly, the role of HSP90 in the suppression of transposon mobility has been identified in other organisms such as nematodes [26] and silkworm [70].

The role of HSP90 in the suppression of TE mobility in nematodes emerged after the exposure of these organisms to environmental stresses accompanied by a reduction in HSP90 levels. In these conditions, the frequency of transposon excisions is amplified in specific loci, supporting the idea that correct HSP90 levels are necessary to keep the transposons silenced. This activity is relevant in both physiological conditions and in the presence of weak environmental stresses [26].

In the silkworm ovary–derived cell line BmN4, HSP90 has a key role in the correct loading of piRNAs precursors onto Piwi proteins [55].

In *Drosophila melanogaster*, it has recently been established that the HSP90/HSP70 chaperone machinery including HSP70/HSP90 organizing protein (Hop), Hsc70-4, and Hsp40, is ordinarily required to load piRNAs onto Ago3 during piRNAs biogenesis (Figure 1). Their displacement from the complex activates the transposable elements following heat shock stress with HSP70 as the main component in this process [70] (Figure 1B).

All of the above reported examples outline the evolutionarily conserved role of HSP90 in the pathways responsible for the silencing of transposons in germ cells to avoid the deleterious effects of their spread in the next generation.

## 3. HSP90, Transposable Elements, and Neurological Diseases

In recent years, a physiological role of transposons in the nervous system of vertebrates and invertebrates is emerging. The studies have highlighted a role for these repetitive sequences in the neuronal genetic plasticity. A paradigmatic example occurs during neuronal development in the human brain where the retrotransposon Line 1 (L1) mobilizes and interrupts protein-coding genes differentially expressed in the brain, contributing to the neuronal mosaicism [71,72]. The transcription factor Sox2 acts to repress L1 transcription in rat adult hippocampal neural stem cells. However, during neuronal differentiation, a decrease in Sox2 expression is correlated with the HDAC1-mediated chromatin remodeling, causing L1 activation [73]. At the same time, Wnt3a and β-catenin signaling induces an increased L1 promoter activity allowing its transcription and retrotransposition [74]. In the adult brain of *Drosophila melanogaster*, new transposon insertions occur in neurons of the mushroom bodies that are the major site for the formation of the memory [75,76]. Interestingly, the transposons’ new insertions in neurons are enriched in genes related to neural functions [76]. Therefore, the attractive hypothesis is that the finely regulated movement of transposable elements appears to be a tool physiologically contributing to brain development and probably to cognitive processes. Accordingly, a dis-regulation of the retrotransposition appears to be related to neuro-developmental diseases such as Rett syndrome [77]. The DNA methyl-binding protein MeCP2, involved in the Rett syndrome, modulates L1 activity during neuronal development with a methylation-related mechanism. L1 performs an elevated frequency of new insertions in MeCP2 knock-out mice and in human brains [73], suggesting a link between elevated retrotranspositions and this neuro-developmental syndrome. Furthermore, other proteins involved in the correct neurological development seem to be strongly related to the transposon silencing pathway in germ cells as demonstrated by the Fragile X protein of *Drosophila melanogaster,* coded by *dFmr1* gene, the Drosophila ortholog of the human *Fmr1* gene, whose mutations are the cause of the Fragile X syndrome [52,78,79]. Fragile X syndrome is one of the most severe hereditary intellectual disabilities in humans. In *Drosophila*, mutations in *dFmr1* gene generate structural and functional neurological alteration with phenotypes superimposable to those observed in human, rendering *Drosophila* a good model to study the syndrome [80]. In 2015, our group established that *dFmr1* has a role in the piRNA pathway in gonads. We demonstrated that dFmr1 genetically and biochemically interacts in gonads and in the nervous system with Ago1 and with Aubergine, the partner of Ago3 in the ping-pong process [52]. Moreover, Ago1 and Ago2 interact with Fmr1 in mammalian somatic tissues and in ovaries [81,82,83].

dFmr1 has been found in complexes with Zinc finger RP-8 (Zfrp8), which participates in the piRNA pathway in *Drosophila* ovaries and is required for the maintenance of hematopoietic, follicle, and germ stem line cells, and is involved in the dFmr1 correct localization. PDCD2, the Zfrp8 homolog in vertebrates, displays similar functions, acting as a chaperone, facilitating protein–protein interactions [84,85]. Interestingly, HSP90, Hsc70-4, Hop, and Piwi have been found in the same complex containing dFmr1 and Zfrp8, corroborating a coordinated role of these proteins in the gonadal piRNA pathway [55,79]. It would be interesting to clarify whether the Zfrp8 complex is restricted to gonads or is present and functional in somatic tissues. On the other hand, there could be the possibility that different proteins might form a similar complex with HSP90, dFmr1, and Hop in the nervous system. It has also been suggested that dFmr1 may have a role in regulating TEs in a specific period during neurological development and that this role may influence synaptic plasticity, learning, and memory during adult life [86]. Other components of somatic piRNA-mediated silencing of transposon such as HSP83 and Shutdown are located in the nervous system, even though they have not been clearly linked to neurological diseases until now.

A role for the TEs’ misregulation has also been suggested for neurodegenerative diseases [87] such as the amyotrophic lateral sclerosis (ALS) caused by functional alterations of the RNA/DNA binding protein TDP-43 [88,89]. Retrotransposition also contributes to neurodegeneration in a *Drosophila* TDP-43 model of ALS. Intriguingly, the TDP-43 protein and dFmr1 physically associate in complexes in vivo in the same ALS *Drosophila* model, and in vitro in neuronal derived cells and regulate common targets [90,91,92,93]. Furthermore, in Hela cells, both HSP90 and HSP70 can be co-immuniprecipitated with TDP-43 [94]. Nevertheless, the molecular mechanism related to the regulation of transposons by TDP-43 has not been completely elucidated. In any case, the unexpected link between TDP-43 and Fmr1 bind these two proteins to HSP90 and HSP90/HSP70 complexes, defining HSP90 as a potential key element in the regulation of transposons in the gonads and most likely in the nervous system.

It would be important to establish how the real consequences are of the transposon activity in the onset of the diseases in which they are activated. This would clarify whether the disease could be associated with the effects on the DNA damage responses caused by transposons or to neurotoxic effects of the elevated amount of TE-related RNAs and proteins in the nervous system.

## 4. Conclusions

Here, we focused on the HSP90-mediated role in the piRNA pathway regulating the transposable elements and preventing genome instability. This specific HSP90 role in the genome stability has been well established in *Drosophila* gonads, affecting the fertility of both sexes [24], and suggests the hypothesis that this role may be implicated in the onset of neurological and neurodegenerative diseases in which the accumulation of misfolded proteins has been demonstrated as in amyotrophic lateral sclerosis (ALS) disease, Alzheimer’s disease (AD), Parkinson’s disease (PD), and front-temporal dementia (FTD). In the above-mentioned diseases, cells exhibit protein aggregates whose composition is specific for each disease such as TDP-43, beta amyloid, tau, and alpha synuclein. The HSP90 chaperone complex targets these aggregates, promoting their correct refolding or their degradation [95,96]. It has been demonstrated that HSP90 plays a role in stabilizing the aberrant aggregating proteins, rendering them more poisonous for the cell [18]. Intriguingly, retrotransposition has been demonstrated to occur in some of the above-mentioned neurological diseases [88,89,96].

Therefore, it would be attractive to investigate a possible link between HSP90 and the control of transposable elements in the nervous system. These studies may clarify events related to the development of cognitive processes and pathogenic mechanisms of neuronal diseases.

## Figures and Tables

**Table 1:** List of genes involved in the piRNA pathway and reported in Figure 1, with the hortologs in humans, their protein domains, and functions. The references refer to the involvement of the reported genes in the piRNA pathway.

Genes	Abbreviation in *Drosophila*	Ortholog in Humans	Protein Type and Domains	Protein Function in the piRNA Pathway	References
*aubergine*	*aub*	*PIWIL1*	Argonaute Protein	primary pathway ping-pong pathway	[45,48,49]
*ago 3*	*ago3*	*PIWIL2*	Argonaute Protein	ping-pong pathway	[45,48,49]
*piwi*	*piwi*	*PIWIL3*	Argonaute Protein	primary pathway	[46]
*armitage*	*armi*	*MOV10L1*	RNA helicases (SDE3)	primary pathway phased piRNA pathway	[50]
*vasa*	*vasa*	*DDX4*	DEAD RNA helicase	primary pathway ping-pong pathway;	[51]
*dFmr1*	*dFmr1*	*FMR1*	Tudor protein KH domain RGG domain	primary pathway ping-pong pathway	[52]
*krimper*	*krimp*	TDRD6	Tudor protein	ping-pong pathway	[53]
*papi*	*papi*	*TDRKH*	Tudor protein KH domain	primary pathway ping-pong pathway	[51]
*qin/kumo*	*qin/kumo*	*TDRD1*	Tudor protein RING domain	primary pathway (dual strand cluster)	[49]
*vreteno*	*vret*	*TDRD15*	Tudor protein RRM domain	primary pathway	[54]
*Yb*	*Yb*	*DDX46*	Tudor protein DEAD RNA helicase	primary pathway (single strand cluster)	[42]
*zucchini*	*zuc*	*PLD6*	Tudor protein nuclease domain	primary pathway phased piRNA pathway	[50]
*hsp83*	*hsp83*	*HSP90*	Heat shock protein	primary pathway ping-pong pathway	[24]
*hsp40*	*hsp40*	*DNAJB5*	Heat shock protein	primary pathway ping-pong pathway	[55]
*hsp70 cognate-4*	*hsc70-4*	*HSPA8*	Heat shock protein cognate	primary pathway ping-pong pathway	[55]
*hsp70/hsp90 organizing protein*	*hop (Sti1)*	*HOP1 (STIP1)*	Cochaperon	primary pathway ping-pong pathway	[55]
*shutdown*	*shu*	*FKBP6*	Cochaperon	primary pathway ping-pong pathway	[56,57]

## Data Availability

Not applicable.
